# Prognostic model for three-year postoperative local recurrence in cutaneous squamous cell carcinoma: a Chinese multicenter cohort study

**DOI:** 10.3389/fonc.2026.1876978

**Published:** 2026-06-15

**Authors:** Yuanhong Liu, Suzheng Zheng, Hao Song, Xuebao Shao, Hao Chen, Wenbo Bu, Guomin Li, Lixiong Gu, Weihao Chen, Jing Fang, Ruzeng Xue, Zhifang Zhai, Yiqun Jiang

**Affiliations:** 1 Hospital for Skin Diseases, Institute of Dermatology, Chinese Academy of Medical Sciences & Peking Union Medical College, Nanjing, China; 2 Dermatology Hospital, Southern Medical University, Guangzhou, China; 3 Department of Dermatology, Affiliated Hospital of Nantong University, Nantong, China; 4 Faculty of Engineering, The University of Sydney, Sydney, Australia; 5 Department of Dermatology, the First Affiliated Hospital, Army Medical University, Chongqing, China

**Keywords:** cutaneous squamous cell carcinoma, nomogram, postoperative recurrence, prognostic model, recurrence-free survival

## Abstract

**Background:**

Cutaneous squamous cell carcinoma (cSCC) carries a risk of postoperative local recurrence, with nearly 90% of events occurring within 3 years; however, prognostic tools tailored to Asian populations remain limited. This study aimed to identify independent risk factors for postoperative local recurrence and develop a nomogram to predict individualized 1-, 2-, and 3-year recurrence probabilities in patients with cSCC.

**Methods:**

Clinicopathological data from 603 patients with cSCC who underwent surgical treatment alone at four Chinese centers were retrospectively analyzed. Patients were randomly divided into a training cohort and a validation cohort at a ratio of 7:3. Univariable and multivariable Cox proportional hazards regression analyses were performed in the training cohort to identify independent predictors of postoperative local recurrence. A nomogram was then constructed based on these independent risk factors. The prognostic performance of the model was evaluated using time-dependent receiver operating characteristic curves, calibration curves, and decision curve analysis. Finally, the total nomogram score was calculated for each patient, and all patients were categorized into low-, intermediate-, and high-risk groups based on the optimal cutoff values. Kaplan–Meier analysis was then performed to compare recurrence-free survival among the different risk groups.

**Results:**

A total of 603 patients were included, with 422 in the training cohort and 181 in the validation cohort. During follow-up, 77 recurrence events were observed. Multivariable Cox regression identified age, tumor size, tumor thickness, histologic differentiation, regional stage, and AJCC stage as independent predictors of recurrence. These predictors were incorporated into a nomogram for individualized recurrence risk estimation. The model demonstrated good discrimination, with area under the curve values ranging from 0.759 to 0.869, along with strong calibration and favorable clinical utility in decision curve analysis. Based on the total nomogram score, patients were stratified into low-risk (<99.0), intermediate-risk (99.0–143.9), and high-risk (≥144.0) groups, with significant differences in recurrence-free survival among the three groups (log-rank p < 0.0001).

**Conclusions:**

This nomogram provides a practical tool for individualized prognostic assessment and may support individualized clinical decision-making for 1-, 2-, and 3-year outcomes ^1^.

## 1 Introduction

Cutaneous squamous cell carcinoma (cSCC) is the second most common type of skin cancer (1). Its global incidence continues to rise, largely driven by population aging and ultraviolet radiation exposure (2). Most cSCCs in exposed sites are associated with chronic ultraviolet exposure, whereas those in non-exposed sites may be related to human papillomavirus (HPV) infection, chronic inflammation, and immune status (3, 4). Beyond local tissue destruction, cSCC may lead to marked disfigurement in exposed areas, adversely affecting patients’ physical function, psychological well-being, and quality of life. Data from the Global Burden of Disease study indicate that both the incidence and prevalence of cSCC in China increased from 1990 to 2019, at a faster rate than the global average (5). Surgery is the most common treatment for cSCC. Although most patients have favorable outcomes after surgical excision, a subset of patients with high-risk clinicopathological features remains at increased risk of local recurrence (6, 7). Even with clear surgical margins, the 5-year cumulative incidences of local and regional recurrence reach 8.7% and 15.3%, with re-recurrence rates of approximately 5.9% (8, 9).

In current cSCC staging systems and most retrospective studies, clinicopathologic factors, including tumor size, depth of invasion, perineural invasion, and poor differentiation, have been associated with adverse prognosis (10–14). However, these systems were primarily developed and validated in Western populations, and prognostic tools tailored to Asian populations remain limited. Meanwhile, previous studies have shown that more than half of cSCC recurrences occur within the first year after diagnosis, and nearly 90% occur within 3 years (6, 15, 16). These findings underscore the critical importance of accurate postoperative risk stratification within the first 3 years after surgery to guide surveillance strategies and identify patients at high risk of recurrence.

Nomograms are graphical tools that integrate multiple variables to provide individualized risk estimates. By translating complex regression models into intuitive visual formats, they improve the interpretability of predictive results and facilitate clinical decision-making (17, 18). Nomograms are widely used in oncology for treatment planning and prognostic assessment, and several high-performing nomograms have been incorporated into the National Comprehensive Cancer Network guidelines, supporting their effectiveness in clinical practice (19, 20). In this study, we aimed to develop a cSCC prognostic nomogram for predicting 1-, 2-, and 3-year postoperative local recurrence probabilities. This model may assist clinicians in individualized postoperative risk stratification and support personalized follow-up management.

## 2 Material and methods

### 2.1 Patient selection

The multicenter data from China were retrospectively collected from four institutions: (1) Hospital for Skin Diseases, Institute of Dermatology, Chinese Academy of Medical Sciences and Peking Union Medical College; (2) Department of Dermatology, First Affiliated Hospital of Army Medical University; (3) Dermatology Hospital of Southern Medical University; (4) Department of Dermatology, Affiliated Hospital of Nantong University. Inclusion criteria were: (1) biopsy-confirmed cSCC with negative surgical margins verified by two pathologists; (2) complete clinical information, including age, sex, tumor size, lesion location, follow-up duration, history of any malignancy, and histopathologic information; (3) patients followed between January 1, 2019 and August 31, 2025. Exclusion criteria were: (1) *in situ* cSCC; (2) received perioperative chemotherapy, radiotherapy, immunotherapy, or photodynamic therapy; (3) missing key clinical variables. After data screening, 603 Chinese patients were included.

### 2.2 Study variables and definitions

Variable selection was based on clinical knowledge, including age, sex, site (exposed vs non-exposed), tumor size, tumor thickness, differentiation, American Joint Committee on Cancer (AJCC) stage, Summary stage (localized, regional, or distant), and prior malignancy history. Tumor thickness was measured as the vertical distance from the top of the granular layer of the epidermis (or the base of an ulcer if ulceration was present) to the deepest point of tumor invasion on digital whole slide images. Summary stage was categorized according to Surveillance, Epidemiology, and End Results (SEER) Summary Stage (2018) as localized (confined to the primary site), regional (spread to subcutaneous fat, neural structures, or regional lymph nodes), or distant metastasis (21). AJCC stage was assigned according to the 8th edition TNM staging system, with no patients classified as stage IV. Exposed sites were defined as the scalp, face, and neck; all other anatomical sites were classified as non-exposed sites. Detailed tumor site classifications are provided in **Supplementary Table 1**.

Negative deep margins were confirmed by pathological review. Only cases in which no tumor cells were identified at the deep resection margin were included in the final analysis. Local recurrence was defined as the reappearance of histologically confirmed cSCC at the original excision site after the initial complete excision. Regional nodal recurrence and distant metastasis were not included. Follow-up time was measured from the date of histopathologic diagnosis to the date of first local recurrence or last follow-up.

### 2.3 Statistical analysis

Categorical and continuous variables were presented as percentages and medians with interquartile ranges (IQRs), respectively, and compared using χ²/Fisher’s exact or Mann–Whitney U/t-tests as appropriate. Data were randomly divided into training and validation cohorts (7:3) for model development and validation. Multicollinearity among variables was evaluated using the variance inflation factor (VIF).

Univariable and multivariable Cox proportional hazards regression analysis were conducted in the training cohort to identify independent risk factors for recurrence. Kaplan–Meier (KM) curves were constructed to estimate recurrence-free survival (RFS), and intergroup differences were compared using the log-rank test. Hazard ratios (HRs) and their 95% confidence intervals (CIs) were calculated. A nomogram was developed to estimate the probability of recurrence within 1, 2, and 3 years for individual patients. Based on the total scores calculated from the recurrence nomogram, the optimal threshold for risk classification was determined. Patients were subsequently categorized into three groups: low-risk, intermediate-risk, and high-risk according to their total scores. KM analyses were used to evaluate differences in RFS among the risk groups.

### 2.4 Validation of the nomograms

To validate the nomogram, its performance was evaluated in both the training and validation cohorts. Model discrimination was assessed using the AUC. Calibration plots were generated to assess the agreement between predicted and observed outcomes, with closer alignment to the 45° reference line indicating better calibration. Bootstrap resampling with 500 iterations was performed in the training cohort to correct for potential model optimism and to provide internal estimates of discrimination and calibration.

**Figure 1:**
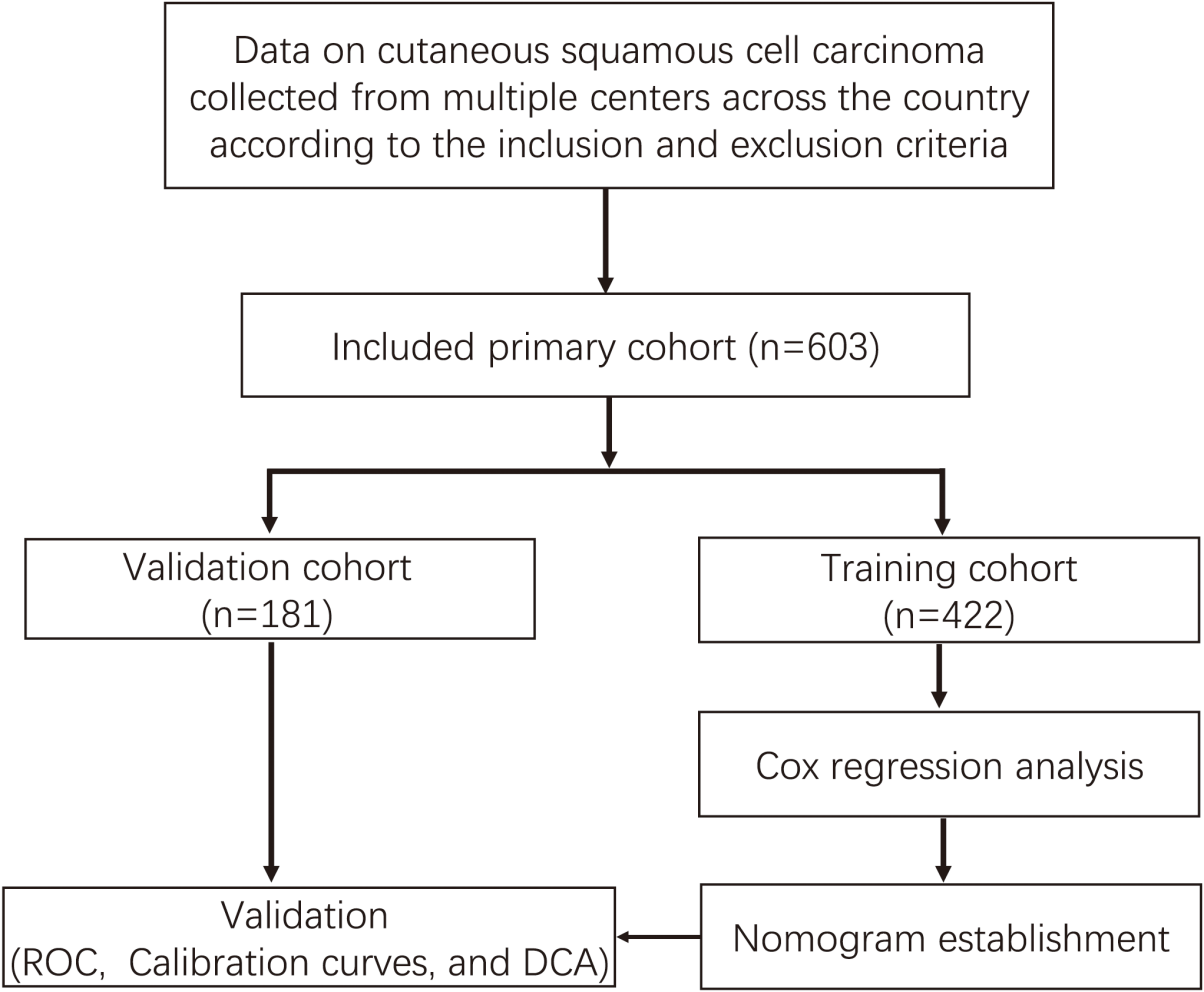
Research flowchart.

DCA was conducted to evaluate the clinical utility of the nomogram. In the DCA plot, the x-axis represents the threshold probability and the y-axis represents the net benefit. The clinical usefulness of the model was assessed by comparing its net benefit with the treat-all and treat-none strategies across a range of threshold probabilities. A higher position of the DCA curve indicates a greater net benefit of the model (22). All statistical analyses were performed using R software (version 4.3.3; https://www.r-project.org/) with the packages survival, rms, survivalROC, and DCA. Two-sided p-values < 0.05 were considered statistically significant. The analytical workflow is illustrated in **Figure 1**.

## 3 Results

### 3.1 Patient characteristics

The Chinese multicenter cohort was randomly divided into a training cohort (n = 422) and a validation cohort (n = 181) using stratified randomization at a 7:3 ratio. The median age was 76 (IQR, 68–84) years in the training cohort and 75 (IQR, 69–82) years in the validation cohort. The median tumor size was 2.0 (IQR 1.1–3.0) cm in both cohorts. In both cohorts, 53.6% and 50.3% of patients were female, and 71.1% and 76.2% of tumors arose from exposed sites. Most tumors were well differentiated (71.8% vs. 72.4%), localized (96.0% vs. 96.7%), AJCC stage I (59.2% vs. 58.6%), and without prior cancer history (97.6% vs. 97.8%). All variables had variance inflation factors (VIFs) <5, indicating no evidence of multicollinearity. The median follow-up time was 24.6 months in the training cohort and 24.3 months in the validation cohort. Baseline clinicopathological characteristics of the training and validation cohorts are summarized in **Table 1**. In addition, baseline clinicopathological characteristics were compared between patients with and without postoperative local recurrence (**Supplementary Table 2**).

**Table 1:** Baseline clinicopathologic characteristics of the training and validation cohorts.

Variable	Whole population		Training cohort		Validation cohort		P-value	VIF
	n	%	n	%	n	%		
Number of patients (n)	603	100.0%	422	70.0%	181	30.0%		
Age	76 (69-83)		76 (68-84)		75 (69-82)		0.941	1.091
Size (cm)	2 (1.1-3)		2 (1.1-3)		2(1.1-3)		0.255	1.499
Thickness(mm)	4.1 (3-6)		4.11 (3-5.8)		4.1(3-6.1)		0.279	1.711
Sex							0.516	1.067
Male	286	47.4%	196	46.4%	90	49.7%		
Female	317	52.6%	226	53.6%	91	50.3%		
Site							0.230	1.009
Exposure	438	72.6%	300	71.1%	138	76.2%		
Non-exposure	165	27.4%	122	28.9%	43	23.8%		
Differentiation							0.962	1.070
Well	434	72.0%	303	71.8%	131	72.4%		
Moderate	97	16.1%	69	16.4%	28	15.5%		
Poor	72	11.9%	50	11.8%	22	12.2%		
Summary stage							0.818	1.126
Localized	580	96.2%	405	96.0%	175	96.7%		
Regional	23	3.8%	17	4.0%	6	3.3%		
AJCC stage (8th edition)							0.907	2.335
I	356	59.0%	250	59.2%	106	58.6%		
II	77	12.8%	55	13.0%	22	12.2%		
III	170	28.2%	117	27.7%	53	29.3%		
History							1.000	1.016
No	589	97.7%	412	97.6%	177	97.8%		
Yes	14	2.3%	10	2.4%	4	2.2%		

### 3.2 Variable screening

**Figure 2:**
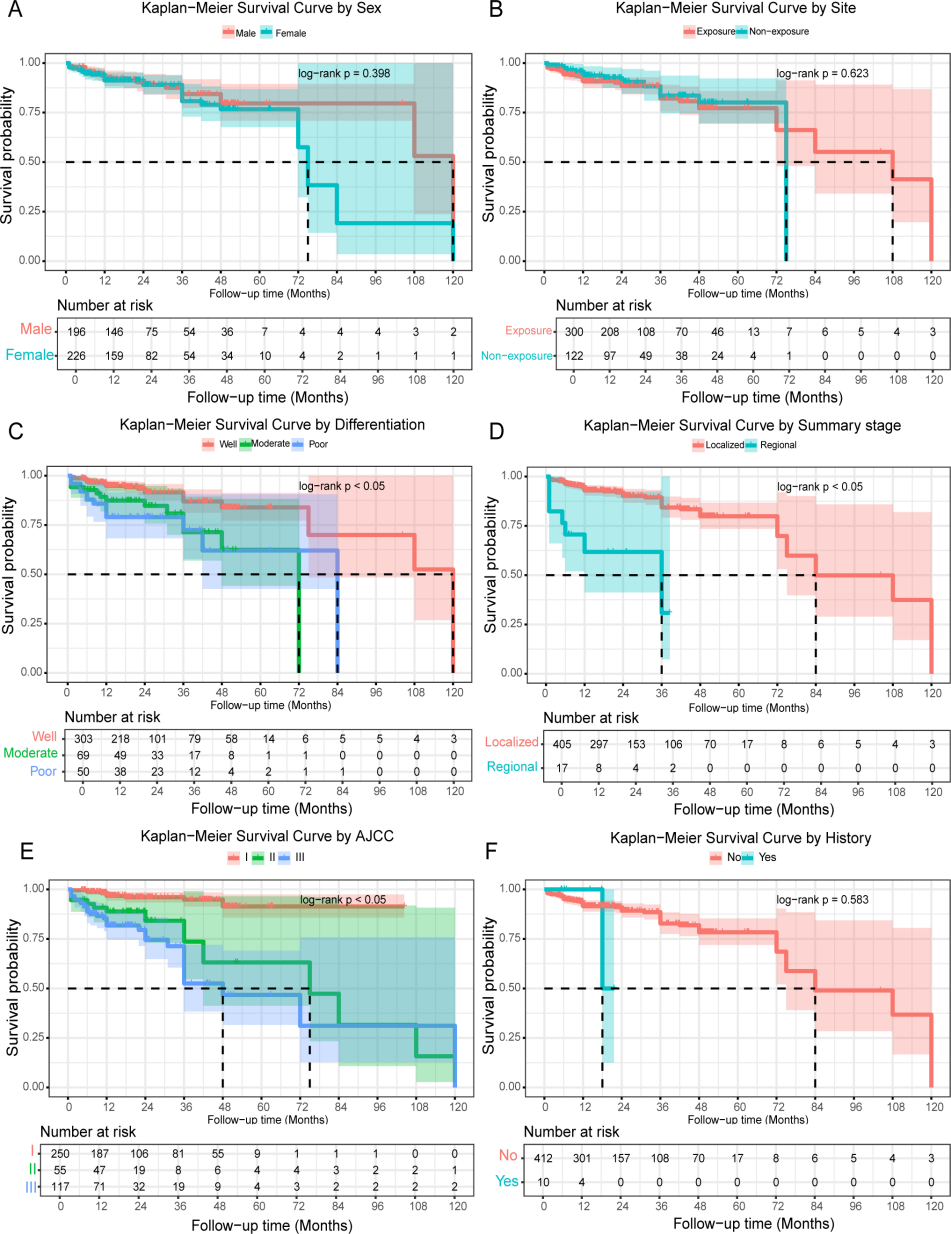
Kaplan–Meier curves for postoperative local recurrence-free survival stratified by clinicopathologic characteristics, including **(A)** sex, **(B)** site, **(C)** differentiation, **(D)** Summary stage, **(E)** AJCC stage, and **(F)** tumor history.

Univariable and multivariable Cox regression analyses were performed to identify predictors of postoperative local recurrence in the training cohort. In the multivariable Cox regression analysis, age, tumor size, tumor thickness, differentiation, Summary stage, and AJCC stage were identified as independent predictors of cSCC recurrence (P < 0.05) (**Table 2**). Increasing tumor size (HR = 1.159, 95% CI: 1.085–1.238) and thickness (HR = 1.153, 95% CI: 1.016–1.308) were associated with a higher recurrence risk, whereas increasing age was associated with a slightly lower recurrence risk (HR = 0.960, 95% CI: 0.939–0.982). Compared with well-differentiated tumors, moderate (HR = 2.353, 95% CI: 1.168–4.739) and poor differentiation (HR = 2.479, 95% CI: 1.179–5.213) were associated with increased recurrence. In addition, regional disease (HR = 2.749, 95% CI: 1.107–6.827) and higher AJCC stage, including stage II (HR = 3.697, 95% CI: 1.537–8.892) and stage III (HR = 2.675, 95% CI: 1.097–6.524), were associated with increased recurrence risk. KM curves according to sex, tumor site, differentiation, Summary stage, AJCC stage, and prior cancer history are shown in **Figure 2**.

**Table 2:** Univariable and multivariable Cox regression analyses of recurrence in the training cohort.

Variable	Univariable analysis		Multivariable analysis	
	HR (95% CI)	P-value	HR (95% CI)	P-value
Age	0.972 (0.954–0.990)	<0.05	0.960 (0.939–0.982)	<0.05
Size (cm)	1.215 (1.158–1.276)	<0.05	1.159 (1.085–1.238)	<0.05
Thickness (mm)	1.203 (1.093–1.325)	<0.05	1.153 (1.016–1.308)	<0.05
Sex				
Male	Reference		Reference	
Female	1.251 (0.736–2.128)	0.408	1.075 (0.601–1.921)	0.808
Site				
Exposure	Reference		Reference	
Non-exposure	0.855 (0.461–1.586)	0.62	1.105 (0.561–2.180)	0.772
Differentiation				
Well	Reference		Reference	
Moderate	2.707 (1.399–5.236)	<0.05	2.353 (1.168–4.739)	<0.05
Poor	3.311 (1.689–6.489)	<0.05	2.479 (1.179–5.213)	<0.05
Summary stage				
Localized	Reference		Reference	
Regional	6.547 (2.911–14.727)	<0.05	2.749 (1.107–6.827)	<0.05
I	Reference		Reference	
II	5.374 (2.351–12.284)	<0.05	3.697 (1.537–8.892)	<0.05
III	7.825 (3.885–15.758)	<0.05	2.675 (1.097–6.524)	<0.05
History				
No	Reference		Reference	
Yes	1.720 (0.234–12.625)	0.594	4.555 (0.583–35.588)	0.148

### 3.3 Nomograms

**Figure 3:**
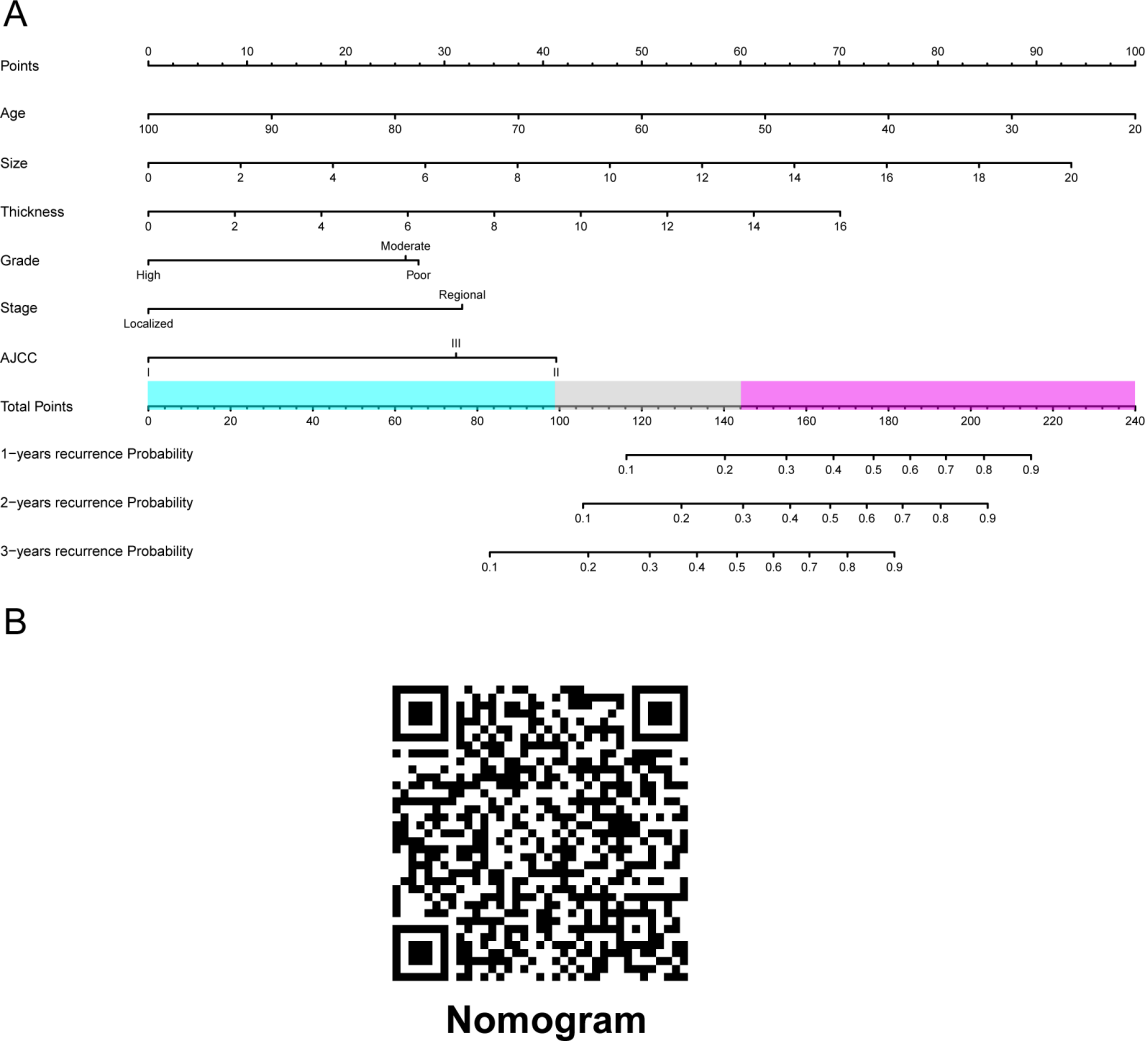
**(A)** Nomogram for predicting 1-, 2-, and 3-year recurrence probabilities. The colored bar in the axis represents risk stratification, with cyan indicating low risk, gray indicating intermediate risk, and purple indicating high risk. **(B)** Web-based dynamic nomogram accessible via QR code.

Independent predictors of tumor recurrence identified through multivariable Cox regression analysis were incorporated into a prognostic nomogram to estimate recurrence probabilities over time (**Figure 3A**). Each variable was assigned a corresponding score, and the total score corresponded to the predicted probability of recurrence on the outcome axis. A web-based dynamic nomogram is accessible via a QR code (**Figure 3B**).

### 3.4 Performance of the nomogram

**Figure 4:**
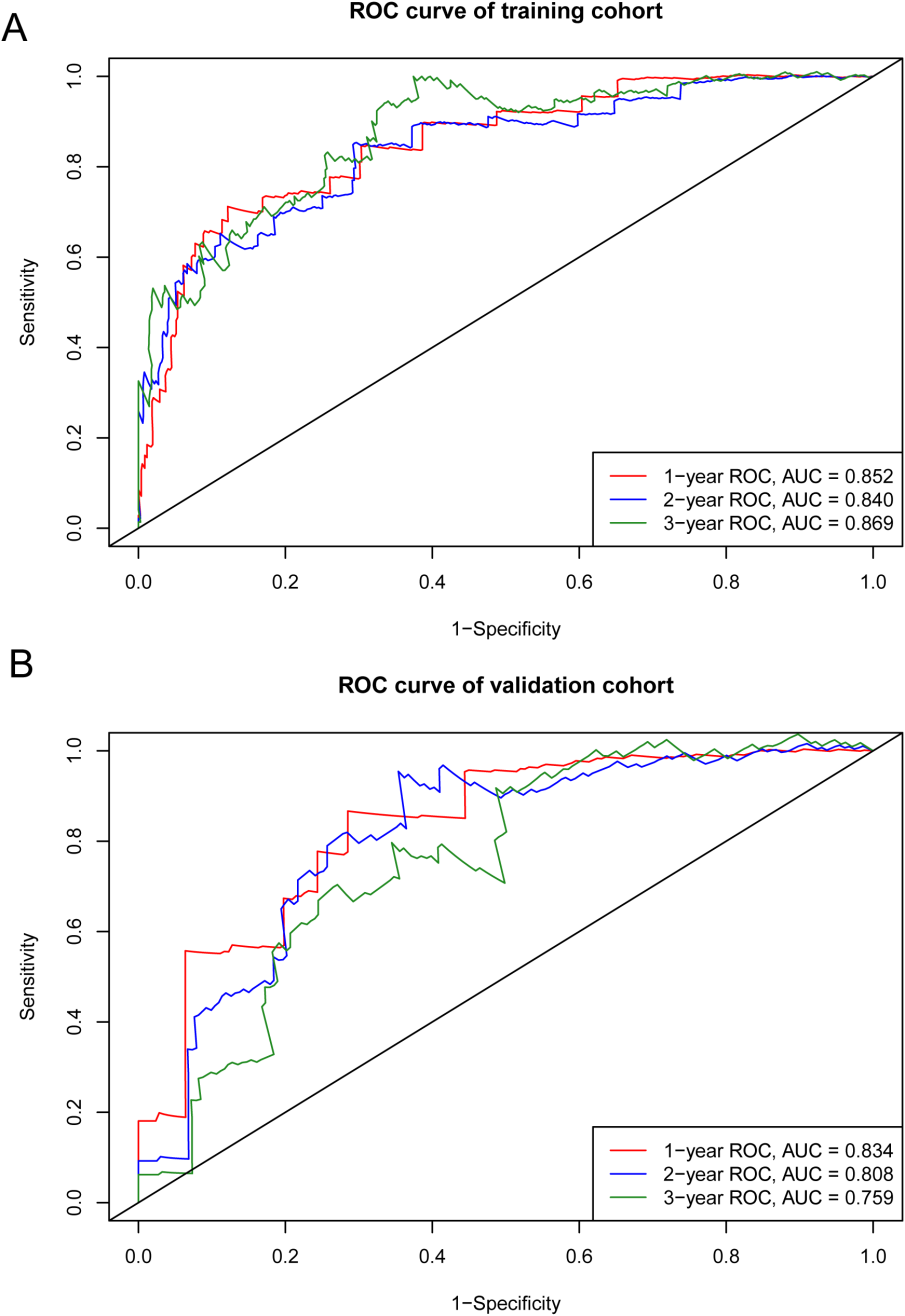
Receiver operating characteristic (ROC) curves demonstrating the discrimination performance of the models. **(A)** Training cohort; **(B)** validation cohort.

**Figure 5:**
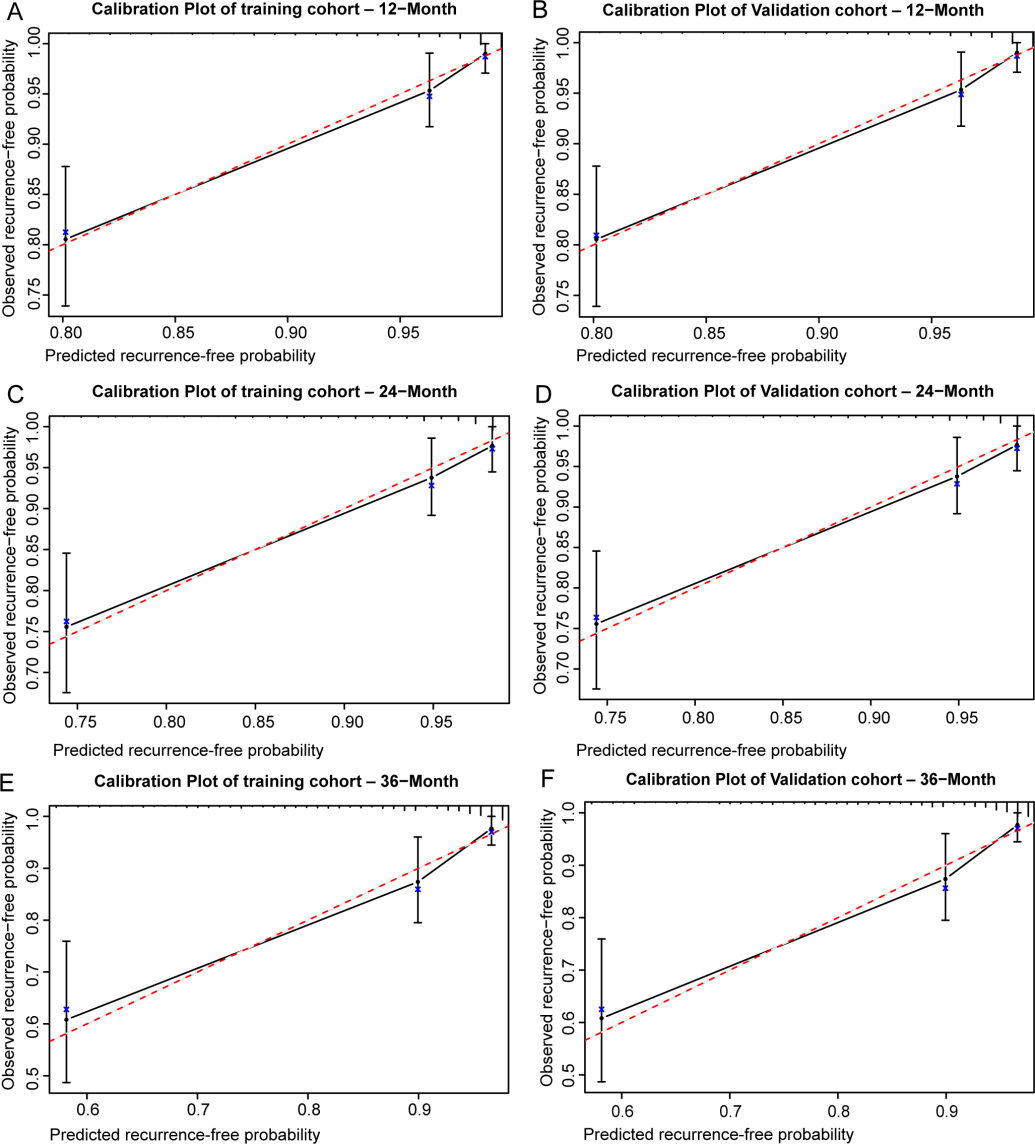
Calibration plots show the agreement between the predicted probabilities based on the nomogram and the observed probabilities ​​for the training set **(A–C)** and validation set **(D–F)**.

**Figure 6:**
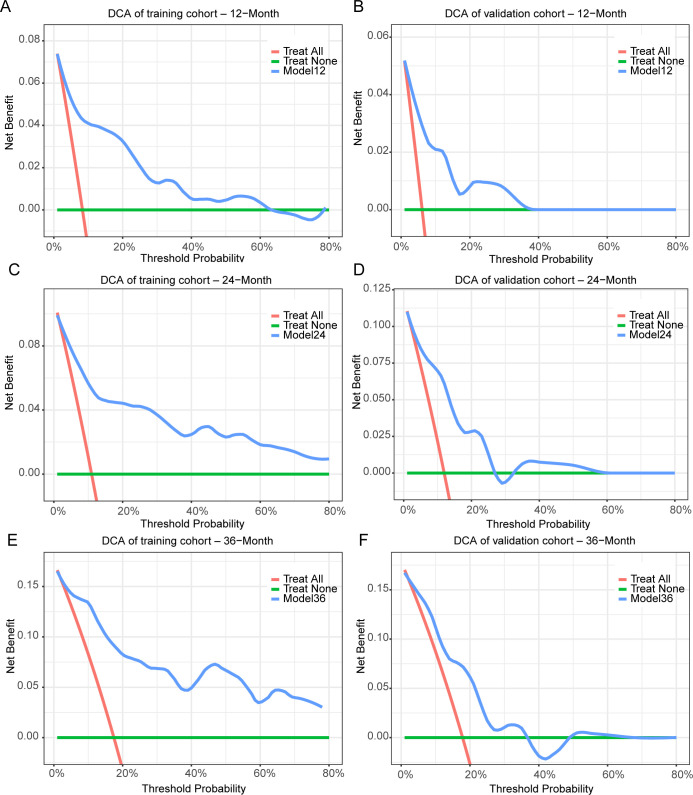
Decision curve analysis (DCA) of the training **(A–C)** and validation **(D–F)** sets of multicenter data.

The nomogram demonstrated good discrimination. The AUCs for predicting 1-, 2-, and 3-year recurrence were 0.852, 0.840, and 0.869 in the training cohort and 0.834, 0.808, and 0.759 in the validation cohort (**Figures 4A, B**). Calibration plots showed good agreement between predicted and observed recurrence probabilities at 12, 24, and 36 months (**Figures 5A–F**), indicating good predictive accuracy and calibration of the model. DCA curves further demonstrated that the nomogram provided net clinical benefit across a range of threshold probabilities at 12, 24, and 36 months (**Figures 6A–F**), supporting its potential clinical utility.

### 3.5 Risk stratification of the recurrence nomogram

**Figure 7:**
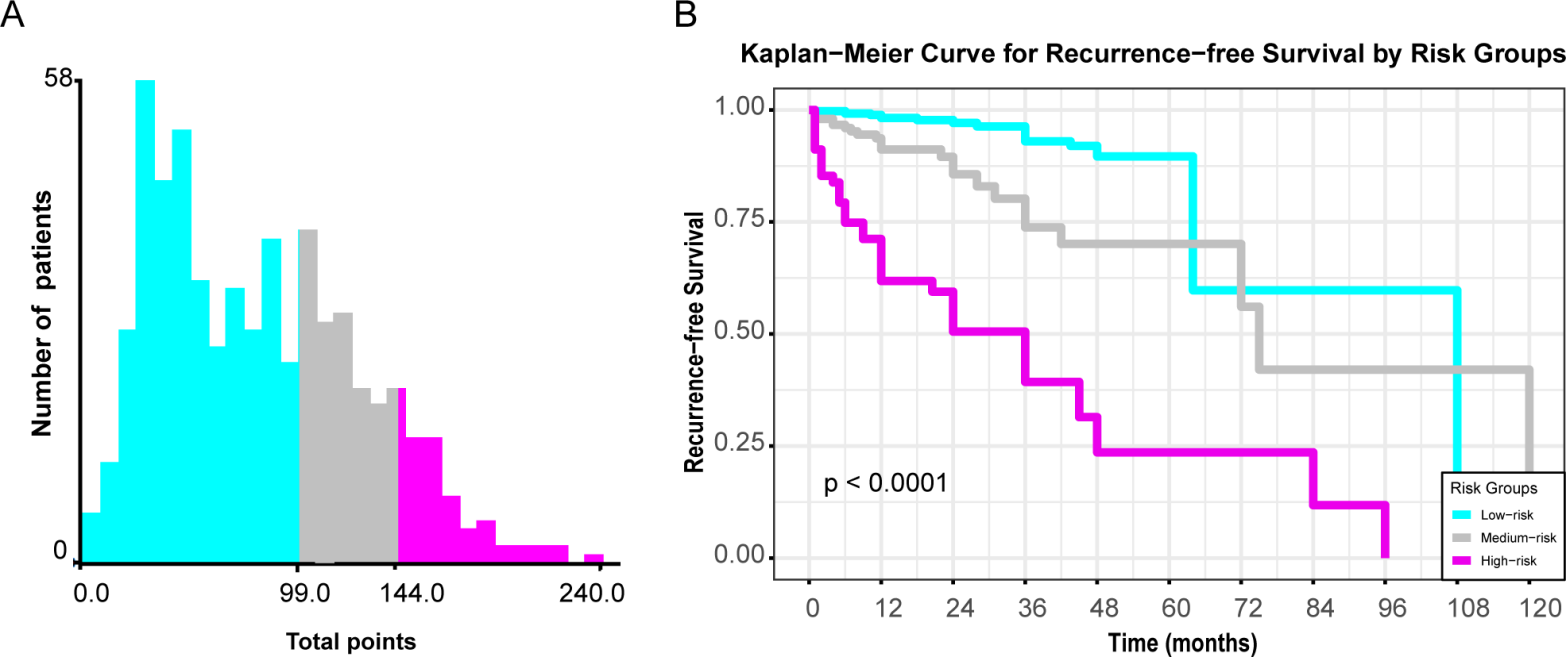
Risk stratification based on the recurrence nomogram. **(A)** Distribution of total scores and corresponding risk group classification. **(B)** Kaplan–Meier curves of recurrence-free survival according to risk groups. .

The total score for each patient was calculated using the recurrence nomogram and used for risk stratification. Patients were categorized into three groups based on predefined cutoff values: low risk (total score < 99.0), intermediate risk (99.0 ≤ total score < 144.0), and high risk (total score ≥ 144.0) (**Figure 7A**). The low-, intermediate-, and high-risk ranges were color-coded on the total score axis of the nomogram for rapid visual risk assessment. KM analysis showed significant differences in RFS among the three risk groups (log-rank p < 0.0001) (**Figure 7B**). These findings indicate that the nomogram-based risk stratification system effectively discriminates patients with different recurrence risks and may serve as a practical tool for individualized postoperative management.

## 4 Discussion

cSCC carries a risk of postoperative local recurrence, which may be associated with increased tumor aggressiveness and treatment difficulty, imposing a substantial health burden. Although the recurrence rate of primary cSCC is generally modest, recurrence risk increases markedly in high-risk patients, with reported local recurrence rates reaching 21.5% in AJCC 8 T4 tumors and 33.5% in Brigham and Women’s Hospital (BWH) T3 tumors (23). Previous studies have investigated prognostic factors for cSCC recurrence, mainly in Western populations (7, 24, 25); however, evidence integrating multiple risk factors for postoperative local recurrence prediction in Asian populations remains limited. Therefore, we developed and validated a prognostic nomogram based on a Chinese cohort to estimate 1-, 2-, and 3-year recurrence probabilities. By integrating readily available clinicopathological variables, the nomogram may facilitate individualized and quantitative risk assessment.

The AJCC staging system plays an important role in prognostic assessment. However, prior studies have shown that adverse outcomes are largely concentrated in patients with T3-stage disease; this stage remains highly heterogeneous, as tumors with different risk profiles may be classified within the same category (12, 26). Furthermore, the AJCC staging system does not fully account for the cumulative effects of multiple high-risk features. Increasing evidence indicates that the coexistence of multiple high-risk factors provides stronger prognostic value than any single factor alone (27). Notably, the AJCC 8th edition staging system also suggests the potential integration of nomograms into future prognostic frameworks (28). Therefore, integrating multiple clinicopathological variables into prognostic models may enable more refined risk stratification compared with reliance on staging systems alone.

In this study, age, tumor size, tumor thickness, differentiation, AJCC stage, and summary stage were identified as independent predictors and incorporated into the nomogram. All variables remained independently associated with recurrence, and no significant multicollinearity was observed (VIF < 5). Among the included variables, AJCC and summary stage showed the greatest contribution to the nomogram, followed by histologic differentiation. Regional stage was associated with a higher recurrence risk than localized stage, and AJCC stage II and stage III were associated with a higher recurrence risk compared with stage I. These findings are in line with the biological behavior of more advanced tumors. Advanced stages typically reflect more aggressive features, including larger tumor size, deeper invasion, perineural invasion, lymphovascular invasion, and nodal involvement and other adverse features, all of which indicate increased tumor aggressiveness and greater tumor burden. Previous studies have demonstrated that tumor depth >6 mm, perineural invasion (≥0.1 mm), and lymphovascular invasion are important predictors of recurrence and metastasis (29), while invasion into subcutaneous fat is associated with an increased risk of local recurrence and disease-specific mortality (27, 30).

Tumor size and tumor thickness were independently associated with recurrence. Increasing tumor diameter has been linked to a higher risk of recurrence (31), particularly when the tumor diameter exceeds 2 cm or depth of invasion exceeds 6 mm (or involves subcutaneous fat) (7, 25, 27, 29, 30, 32, 33). In addition to tumor size and thickness, histologic differentiation also demonstrated prognostic significance. Both moderate and poor differentiation were associated with increased recurrence risk. Although only poor differentiation has long been regarded as a marker of aggressive tumor behavior (7, 32, 34, 35), histologic grading is subject to interobserver variability, which may limit its prognostic stability (36). These findings suggest that histologic differentiation should be interpreted with caution, as moderately differentiated tumors may not necessarily represent a low-risk group and should be evaluated alongside other clinicopathologic factors.

Age showed a slight inverse association with recurrence in our cohort. Previous studies reported that elderly patients tend to present with high-risk tumor features such as larger tumor size, poor differentiation, and deeper tumor invasion, which are generally associated with an increased risk of recurrence (37–41). In our cohort, this inverse finding may be explained by the relatively high median age of the cohort (76 years) and the lack of individual-level mortality data. As a result, older patients may have been more likely to die before recurrence was observed. In the absence of individual-level competing event data, a standard Cox model may underestimate recurrence risk in older patients (42, 43).

No significant associations were observed for sex or sun-exposure status in the Chinese cohort. Previous studies in Western populations have reported sex-specific anatomical distributions, with men more commonly affected at head and neck sites and women at the lower extremities, where recurrence risk has been reported to be relatively higher (44, 45). The absence of such associations in our study may be explained by several factors. First, the Chinese cohort predominantly comprised conventional cutaneous cSCC arising from sun-exposed sites. Although this distribution reflects the typical epidemiology of cSCC, the relative lack of anatomical diversity may have limited the ability to detect potential sex- or exposure-related differences. Second, these associations may be population-specific and influenced by differences in epidemiological and anatomical distributions. Third, Fitzpatrick skin types III–IV are more prevalent in Chinese populations and exhibit lower sensitivity to ultraviolet radiation compared with types I–II, which may attenuate UV-related anatomical variation (46). However, this hypothesis requires further validation in larger and more diverse populations.

Notably, due to differences in tumor microenvironment, lymphatic drainage, and biological behavior across anatomical sites, cSCC exhibits substantial anatomical heterogeneity. Conventional cSCC is strongly associated with chronic ultraviolet exposure, whereas tumors arising at mucocutaneous genital sites are more frequently linked to HPV infection, chronic inflammation, and immune status (3, 4). Site-specific analysis showed that cSCC has a higher incidence and better prognosis than mucocutaneous genital SCC, with 5-year survival rates of approximately 93%–97% versus 71%–75% (44). As our multicenter cohort predominantly comprised conventional cutaneous cSCC, the model may not be directly applicable to mucocutaneous junction sites, such as vulvar and genital cSCC.

Although the current nomogram was constructed using routinely available clinicopathological variables, future model refinement may benefit from the integration of molecular biomarkers. Previous genomic studies have shown that molecular alterations are involved in cSCC carcinogenesis and aggressive tumor behavior. For example, NOTCH1 mutations have been reported as an early event in cutaneous squamous cell carcinogenesis, while TP53 and KMT2D mutations were found at higher frequencies in metastatic cSCC than in primary tumors (47, 48). Genomic alterations involving CDKN2A, NOTCH1/2, FAT1, RAS and EGFR have also been implicated in the molecular pathogenesis and heterogeneity of cSCC (49). In addition, liquid biomarkers, such as baseline serum IFN-γ and cfDNA, may provide complementary information for dynamic monitoring in advanced or immunotherapy-treated cSCC (50). However, their prognostic value for postoperative recurrence prediction remains insufficiently validated. Future studies integrating molecular biomarkers with clinicopathological variables may further improve the biological interpretability and predictive performance of recurrence prediction models for cSCC.

In this study, a prognostic nomogram was developed and validated to predict 1-, 2-, and 3-year local recurrence probabilities in patients with cSCC based on an Asian cohort. The model demonstrated good prognostic performance in both the training and validation cohorts. By integrating readily available clinicopathological variables, the nomogram may help stratify patients according to postoperative local recurrence risk and guide individualized follow-up strategies. High-risk patients may benefit from closer surveillance and additional evaluation when clinically indicated, whereas low-risk patients may continue routine follow-up, thereby supporting more efficient allocation of clinical resources.

## 5 Limitations

This study has several limitations. First, the retrospective design may introduce selection and information biases. Second, given the relatively elderly study population, competing mortality may have influenced recurrence estimation. Although patient survival status was available during follow-up, detailed and reliable cause-of-death information could not be obtained. Therefore, deaths unrelated to cSCC recurrence could not be accurately classified as competing events, which limited the use of formal competing-risk analysis. This may have biased recurrence estimates, particularly among older patients who died before recurrence was observed. Future prospective studies with standardized cause-specific follow-up data are needed to validate the model using competing-risk methods. Third, although the model underwent split-sample validation, this still represents internal rather than true external validation. Because all cohorts were derived from Chinese institutions, the applicability of the nomogram to other geographic regions and populations remains uncertain. Independent external validation in geographically diverse and prospective cohorts is needed before clinical implementation. Fourth, several clinically relevant prognostic variables were not fully incorporated. Although perineural invasion and lymphovascular invasion were recorded in this cohort, only a small number of patients had these features, which limited their stability as independent variables in multivariable modeling. Previous malignancy history was included in the model, but other comorbid conditions, such as lupus erythematosus or burn scars, were too rare to be modeled reliably. Immunosuppression status was also not included because detailed records were not consistently available. Future prospective studies should systematically collect perineural invasion, lymphovascular invasion, immunosuppression-related information, and other clinically relevant risk factors to evaluate their independent contribution to recurrence prediction. Finally, although all included cases had pathologically confirmed negative deep margins, the exact margin distance was not systematically recorded. Therefore, the association between close margins and postoperative recurrence could not be assessed, and should be evaluated in future prospective studies.

## Data Availability

The data that support the findings of this study are available from the corresponding authors upon reasonable request.
