# Supplementary material for: Prognostic model for three-year postoperative local recurrence in cutaneous squamous cell carcinoma: a Chinese multicenter cohort study
Source: Front Oncol. 2026 Jun 15;16:1876978. doi: 10.3389/fonc.2026.1876978 (PMC13310780; doi:10.3389/fonc.2026.1876978)
Supplement: Supplementary file 1 [file Table1.docx]

***Supplementary Material***

Supplementary Methods

Supplementary Table S1. Anatomical distribution of Chinese cohort

|  | Primary Site | n (% of total cohort) |
| --- | --- | --- |
| Exposure  (n=438) | Scalp | 80 (13.27) |
|  | Face | 353 (58.54) |
|  | Neck | 5 (0.83) |
| Non-exposure  (n=165) | Hand | 39 (6.47) |
|  | Upper extremity | 21 (3.48) |
|  | Trunk | 23 (3.81) |
|  | Lower extremity | 64 (10.61) |
|  | Foot | 18 (2.98) |

Supplementary Table S2. Baseline characteristics of patients with and without postoperative local recurrence

| Variable | Non-recurrence patients  (n = 526) | Recurrence patients  (n = 77) | p-value |
| --- | --- | --- | --- |
| Age, years | 77 (70–84) | 71 (63–77) | <0.001 |
| Tumor size, cm | 1.6 (1.0–3.0) | 3.0 (2.0–4.5) | <0.001 |
| Tumor thickness, mm | 4.0 (3.0–5.8) | 5.1 (3.5–7.2) | 0.001 |
| Sex, n (%) |  |  | 0.625 |
| Male | 247 (47.0) | 39 (50.6) |  |
| Female | 279 (53.0) | 38 (49.4) |  |
| Tumor site, n (%) |  |  | 0.571 |
| Exposed sites | 380 (72.2) | 58 (75.3) |  |
| Non-exposed sites | 146 (27.8) | 19 (24.7) |  |
| Histological differentiation, n (%) |  |  | <0.001 |
| Well | 397 (75.5) | 37 (48.1) |  |
| Moderate | 77 (14.6) | 20 (26.0) |  |
| Poor | 52 (9.9) | 20 (26.0) |  |
| Summary stage, n (%) |  |  | <0.001 |
| Localized | 514 (97.7) | 66 (85.7) |  |
| Regional | 12 (2.3) | 11 (14.3) |  |
| AJCC stage, 8th edition, n (%) |  |  | <0.001 |
| I | 337 (64.1) | 19 (24.7) |  |
| II | 59 (11.2) | 18 (23.4) |  |
| III | 130 (24.7) | 40 (51.9) |  |
| History of prior malignancy, n (%) |  |  | 0.091 |
| No | 516 (98.1) | 73 (94.8) |  |
| Yes | 10 (1.9) | 4 (5.2) |  |

Abbreviations: AJCC, American Joint Committee on Cancer

| Supplementary Table S3. Multivariable Cox regression analysis of LASSO-selected variables in the training cohort | | | |
| --- | --- | --- | --- |
| Variable | HR | 95% CI | p-value |
| Age | 0.960 | 0.940-0.981 | <0.05 |
| Size (cm) | 1.159 | 1.089-1.235 | <0.05 |
| Thickness (mm) | 1.153 | 1.016-1.308 | <0.05 |
| Differentiation |  | | |
| Well | Reference | | |
| Moderate | 2.314 | 1.153-4.645 | <0.05 |
| Poor | 2.423 | 1.183-4.961 | <0.05 |
| Summary stage |  | | |
| Localized | Reference | | |
| Regional | 2.767 | 1.112-6.886 | <0.05 |
| AJCC stage, 8th edition |  | | |
| I | Reference | | |
| II | 3.772 | 1.577-9.019 | <0.05 |
| III | 2.665 | 1.093-6.499 | <0.05 |
| History |  | | |
| No | Reference | | |
| Yes | 4.601 | 0.590-35.878 | 0.145 |

Abbreviations: AJCC, American Joint Committee on Cancer

Supplementary Table S4. Sensitivity analysis for the inclusion of AJCC stage and Summary stage.

| Model | C-index | AIC | P value |
| --- | --- | --- | --- |
| Full model (AJCC Stage + Summary Stage) | 0.844 | 497.59 | — |
| AJCC Stage only | 0.841 | 499.66 | <0.05 |
| Summary Stage only | 0.848 | 502.66 | <0.05 |
| *P values from likelihood ratio test comparing each reduced model against the full model. | | | |


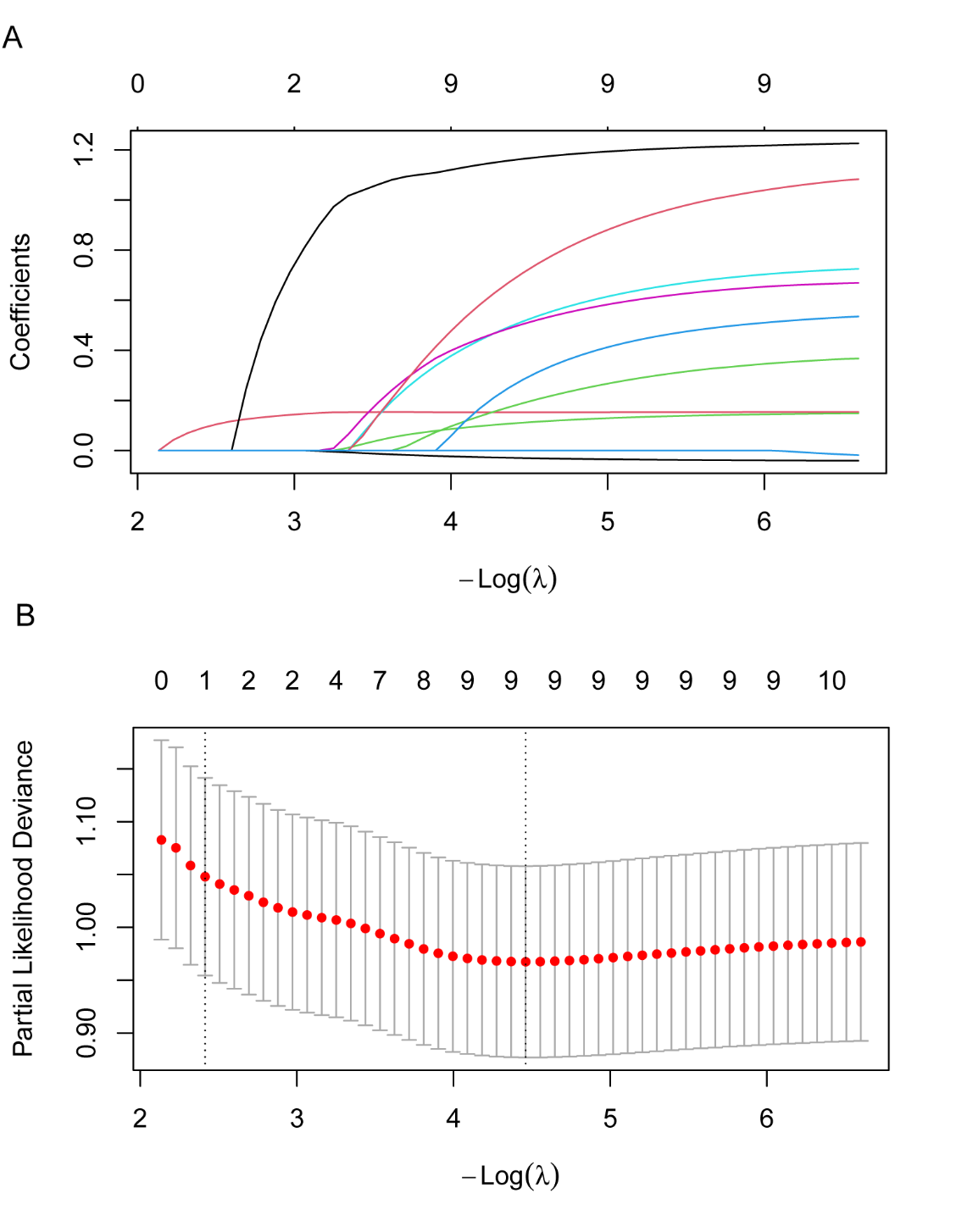


Supplementary Figure S1. Variable selection using LASSO regression.

(A) LASSO coefficient paths. (B) Cross-validation curve for optimal λ selection.
